# African-American Race Predicts 1-Year Cognitive Decline Among Adults Without Moderate Dementia

**DOI:** 10.1093/geroni/igab046.2378

**Published:** 2021-12-17

**Authors:** Niser Babiker, Alan Gonzalez, Jovany Soto, Chengjian Shi, Andrey Rzhetsky, Megan Huisingh-Scheetz

**Affiliations:** 1 University of Chicago, Chicago, Illinois, United States; 2 Illinois Institute of Technology, Orland Park, Illinois, United States; 3 University of Chicago, chicago, Illinois, United States; 4 University of Chicacgo, Chicago, Illinois, United States

## Abstract

Previous literature shows conflicting conclusions about the association between race and cognitive decline, particularly in early impairment. In this study, we aimed to test whether race predicted 1-year change in Montreal Cognitive Assessment (MoCA) score among older adults without moderate-severe dementia. We secondarily explored whether multimorbidity, polypharmacy, depressed mood, antidepressant use, body composition, or frailty changed the association. We analyzed data (n=122) from predominantly African American (AfA, 78.7%) community-dwelling older adults from the south side of Chicago. Participants underwent baseline and 1-year MoCA testing. Age, gender, race, education, monthly income, co-morbidities (Charlson Comorbidity Index), medication use (<5 vs ≥5), depression (PHQ-2), proportion lean mass (DEXA), and the frailty phenotype (range 0-5) were collected at baseline. In a multivariate linear model, we regressed 1-year MoCA score on baseline MoCA score, race, and demographics and then evaluated the impact of each covariate added separately to the model on the race-cognition relationship. The mean MoCA score at baseline was 25.2+/-0.2 (range 18-30) and 41.0% of participants experienced ≥1 point MoCA decline at 1 year. After adjusting for demographics, AfAs experienced a greater 1-year MoCA decline (β= -1.3, p=0.04) compared to other races. The effect size was unchanged after adjusting for multimorbidity and polypharmacy (β= -1.3, p=0.04), attenuated slightly after adjusting for frailty (β= -1.2, p=0.06), depressed mood (β= -1.2, p=0.05), lean mass (β= -1.2, p=0.04), and attenuated notably after adjusting for antidepressant use (β= -1.0, p=0.11). Findings support the need to further explore racial differences in cognitive decline and potentially related anti-depressant underuse.

